# Supplementary material for: Iterative improvement in the automatic modular design of robot swarms
Source: PeerJ Comput Sci. 2020 Dec 7;6:e322. doi: 10.7717/peerj-cs.322 (PMC7924708; doi:10.7717/peerj-cs.322)
Supplement: Supplemental Information 3 [file peerj-cs-06-322-s003.zip › argos3/doc/api/standalone/a00438_source.html]

ARGoS: plugins/robots/foot-bot/control\_interface/ci\_footbot\_distance\_scanner\_sensor.cpp Source File


- Main Page
- Related Pages
- Namespaces
- Classes
- Files

- File List
- File Members

# plugins/robots/foot-bot/control\_interface/ci\_footbot\_distance\_scanner\_sensor.cpp

Go to the documentation of this file.

```
00001 
00007 #include "ci_footbot_distance_scanner_sensor.h"
00008 
00009 #ifdef ARGOS_WITH_LUA
00010 #include <argos3/core/wrappers/lua/lua_utility.h>
00011 #endif
00012 
00013 namespace argos {
00014    
00015   /****************************************/
00016   /****************************************/
00017 
00018   const CCI_FootBotDistanceScannerSensor::TReadingsMap& CCI_FootBotDistanceScannerSensor::GetReadingsMap(){
00019     return m_tReadingsMap;
00020   }
00021 
00022   /****************************************/
00023   /****************************************/
00024 
00025   const CCI_FootBotDistanceScannerSensor::TReadingsMap& CCI_FootBotDistanceScannerSensor::GetShortReadingsMap(){
00026     return m_tShortReadingsMap;
00027   }
00028 
00029   /****************************************/
00030   /****************************************/
00031 
00032   const CCI_FootBotDistanceScannerSensor::TReadingsMap& CCI_FootBotDistanceScannerSensor::GetLongReadingsMap(){
00033     return m_tLongReadingsMap;
00034   }
00035 
00036    
00037 #ifdef ARGOS_WITH_LUA
00038   void CCI_FootBotDistanceScannerSensor::CreateLuaState(lua_State* pt_lua_state) {
00039     CLuaUtility::OpenRobotStateTable (pt_lua_state, "distance_scanner");
00040     CLuaUtility::StartTable          (pt_lua_state, "short_range"     );
00041     CLuaUtility::EndTable            (pt_lua_state                    );
00042     CLuaUtility::StartTable          (pt_lua_state, "long_range"      );
00043     CLuaUtility::EndTable            (pt_lua_state                    );
00044     CLuaUtility::CloseRobotStateTable(pt_lua_state                    );
00045   }
00046 #endif
00047 
00048   /****************************************/
00049   /****************************************/
00050 
00051 #ifdef ARGOS_WITH_LUA
00052   void CCI_FootBotDistanceScannerSensor::ReadingsToLuaState(lua_State* pt_lua_state) {
00053     lua_getfield(pt_lua_state, -1, "distance_scanner");
00054     CLuaUtility::StartTable(pt_lua_state, "short_range");
00055     int nCounter = 1;
00056     for(TReadingsMap::iterator it = m_tShortReadingsMap.begin();
00057         it != m_tShortReadingsMap.end(); ++it) {
00058       CLuaUtility::StartTable(pt_lua_state, nCounter);
00059       CLuaUtility::AddToTable(pt_lua_state, "angle",    it->first);
00060       CLuaUtility::AddToTable(pt_lua_state, "distance", it->second);
00061       CLuaUtility::EndTable(pt_lua_state);
00062       ++nCounter;
00063     }
00064     CLuaUtility::EndTable(pt_lua_state);
00065     CLuaUtility::StartTable(pt_lua_state, "long_range");
00066     nCounter = 1;
00067     for(TReadingsMap::iterator it = m_tLongReadingsMap.begin();
00068         it != m_tLongReadingsMap.end(); ++it) {
00069       CLuaUtility::StartTable(pt_lua_state, nCounter);
00070       CLuaUtility::AddToTable(pt_lua_state, "angle",    it->first);
00071       CLuaUtility::AddToTable(pt_lua_state, "distance", it->second);
00072       CLuaUtility::EndTable(pt_lua_state);
00073       ++nCounter;
00074     }
00075     CLuaUtility::EndTable(pt_lua_state);
00076     lua_pop(pt_lua_state, 1);
00077   }
00078 #endif
00079 
00080 
00081   /****************************************/
00082   /****************************************/
00083 
00084 }
```

---

Generated on 10 Jul 2018 for ARGoS by 
 1.6.1
